# Diagnostic Utility of a Multiplex PCR Assay in Detecting Common Mutations of the α‐Globin Gene in α‐Thalassemia

**DOI:** 10.1155/anem/9991675

**Published:** 2025-10-14

**Authors:** Si Nae Park, Jin Roh, Jin-Tae Kim, Min-Jung Song

**Affiliations:** ^1^ Department of BT Research Institute, U2Bio Co. Ltd., Seoul, Republic of Korea; ^2^ U2Bio (Thailand) Co. Ltd., Bangkok, Thailand; ^3^ Clinical Research Center, Jang-Won Medical Foundation, Seoul, Republic of Korea

**Keywords:** α-globin, α-thalassemia, *HBA*, hemoglobin disorder, large deletions, multiplex PCR assay

## Abstract

Alpha‐thalassemia is a hereditary hemoglobin disorder characterized by reduced or absent α‐globin gene, and its severity is associated with the number of affected alleles. Several methods are available for detecting α‐thalassemia, such as multiplex ligation‐dependent probe amplification (MLPA) and PCR‐based hybridization strip assay. Multiplex PCR offers a faster, more convenient, and cost‐effective alternative. In this study, we aimed to optimize a current PCR‐based method for α‐thalassemia screening and evaluate its utility using clinical samples. We also investigated the prevalence and spectrum of common mutations responsible for α‐thalassemia in Thailand and Korea. A total of 1261 samples from Thailand, 560 samples from different ethnic groups residing in Korea, and 300 samples from native Koreans were collected and tested. The concordance rate between the data collected in Thailand and in this study was 99.92%. Further, approximately 5.9% of the non‐Korean individuals living in Korea were identified as healthy carriers, whereas no mutations were observed in Koreans. Comparing the data with MLPA or Sanger sequencing data showed 100% agreement rate in both cases. We successfully developed a PCR method for the diagnosis of α‐thalassemia that is fast, less labor‐intensive, and cost‐effective. Given the performance results of this method, it has great potential for application in α‐thalassemia diagnosis.

## 1. Introduction

Alpha (α)‐thalassemia (OMIM 604131) is one of the most prevalent inherited hemoglobin disorders and is characterized by reduced or absent α‐globin chain synthesis [1, 2]. Normal individuals have two α‐globin genes on each chromosome. The severity of the condition is correlated with the number of affected α‐globin alleles. Individuals with one α‐globin gene deletion are clinically and hematologically silent carriers, while those with two α‐globin gene deletions express the α‐thalassemia trait, which results in very mild microcytic hypochromic anemia. When three α‐globin genes become inactive, the affected individual is said to have moderate anemia and a hereditary disorder known as the hemoglobin (Hb) H disease. The most severe form of α‐thalassemia is the Hb Bart’s hydrops fetalis syndrome, in which the individual lacks all α‐globin genes [3–5].

Double‐gene deletions *cis* of α‐thalassemia are clinically important because homozygosity or compound heterozygosity for such deletions lead to fetal death shortly after birth [6]. Infants who do not inherit α‐globin genes from their parents are said to have Hb Bart’s hydrops fetalis syndrome. Further, both ‐‐^FIL^ and ‐‐^THAI^ mutations result in the deletion of embryonic zeta (*ζ*)‐ and α‐globin genes, and homozygotes that are ‐‐^FIL^/‐‐^FIL^ and ‐‐^THAI^/‐‐^THAI^ usually die very early in gestation because they cannot produce embryonic Hb. However, compound heterozygotes (e.g., ‐‐^FIL^/‐‐^SEA^) mutations exhibit the classic form of Hb Bart’s hydrops fetalis syndrome [7, 8].

α‐Thalassemia is more frequently caused by deletion than nondeletional mutations. Some of these nondeletional mutations may cause a more severe reduction in α‐chain synthesis than a single α‐gene deletion in the chromosome. These mutations affect gene expression, mRNA splicing, and degradation. Termination codon mutations, such as Hb Constant Spring (Hb CS) and Hb Paksé (Hb PS), transform the stop codon at position 142 into a coding sequence. Such mutations also result in an elongated α‐chain and a highly unstable hemoglobin variant with a low expression level [3, 9, 10]. Hb CS (c.427T > C p.X143Gn) and Hb PS (c.429A > T p.X143Leu) mutations are the most common nondeletional mutation in the Oriental population, and misdiagnosis of these mutations is common in routine hematological analyses [1, 10–12].

Furthermore, the common types of deletional mutations vary across regions. The prevalence of α‐thalassemia is high in Southeast Asia, the Mediterranean region, and Africa, while the ‐‐^SEA^ type mutation is common in Southeast Asia, and the ‐‐^FIL^ type is relatively common in the Philippines. Additionally, ‐‐^MED^ and ‐(α)^20.5^ deletions are relatively common in the Mediterranean region, and deletions of a single α‐gene, such as −α^3.7^ and −α^4.2^, are common in African Americans, as well as in Saudi Arabia, India, Thailand, Papua New Guinea, and Melanesia [3].

Prenatal screening and carrier detection are important for populations where α‐thalassemia is prevalent. The main reason for prenatal diagnosis is to avoid pregnancies affected by Hb Bart’s hydrops fetalis syndrome, as repeated pregnancies of this nature may pose a considerable risk to the mother [10]. Reported complications during pregnancies involving Hb Bart’s fetus include preeclampsia, poly‐/oligohydramnios, hemorrhage, anemia, and sepsis. These complications constitute a significant basis for counseling and discussions regarding the option of selective abortion [1, 9, 13, 14]. Additionally, individuals with α‐thalassemia traits usually exhibit a normal Hb concentration or mild anemia and can still donate blood. However, the impact of thalassemia trait donors on transfusion outcomes for both donors and recipients requires further study, as the post‐transfusion RBC survival may be lower than in normal cases. For the efficacy of the transfusion, screening the donors for α‐thalassemia is recommended in regions with a high prevalence of the disease [15–17]. Furthermore, screening for α‐thalassemia and its related complications may reduce the morbidity and mortality of aging patients with the condition [18].

Although α‐thalassemia is highly prevalent in the Mediterranean [19, 20], Southeast Asia [12, 21–23], and Africa, screening is not limited to these areas. Over the past few decades, massive migrations have occurred, and the prevalence of α‐thalassemia has increased worldwide. Thus, it is important for clinicians in Northern Europe, North America [24, 25], and South Korea [26, 27] to pay attention to this rare disease.

Southern blot analysis, which is labor‐intensive and expensive, was previously the standard procedure for the molecular characterization of α‐thalassemia. Recently, the diagnosis of α‐thalassemia has been facilitated by Sanger sequencing and multiplex ligation‐dependent probe amplification (MLPA). However, this technique is also expensive and requires additional test steps compared to PCR‐only methods. As PCR‐based assays are more rapid, less expensive, safer, and easier to interpret, several PCR‐based methods have been developed for the diagnosis of different subsets of α‐thalassemia. However, the diagnosis of α‐thalassemia using PCR‐based tests is problematic owing to the high GC nucleotide content and high degree of homology within the α‐gene cluster [28, 29]; some of these methods showed poor reproducibility or high interference when multiplexed.

This study aims to optimize a current multiplex PCR assay [2, 4, 6, 28, 29] into a three‐tube multiplex PCR assay to the end of avoiding the competing reactions of each primer and improve interpretation. We also aimed to compare the analytical performance of the modified multiplex PCR assay with Sanger sequencing to determine its diagnostic utility for the detection of α‐thalassemia. Furthermore, we aim to investigate the prevalence and spectrum of the common α‐globin gene mutations responsible for α‐thalassemia in Thailand and Korea.

## 2. Materials and Methods

### 2.1. Subjects

From 2017 to 2022, we collected a total of 2121 samples, of which 1261 were genomic DNA samples from U2Bio Co. Ltd. in Thailand (South Bangkok and Northeastern Thailand) and subjected them to the α‐thalassemia test based on a multiplex PCR using in‐house primers from U2Bio Co. Ltd. (Bangkok, Thailand). In Korea, 300 and 560 blood samples from Koreans and different ethnic groups living in Korea, respectively, were collected from Jang‐won Medical Foundation to determine the proportion of patients with α‐thalassemia among the asymptomatic population. The samples were then anonymized by assigning them random numbers and, thereafter, transferred to the BT Research Institute of U2Bio (Seoul, Korea) for analysis. Hematological test data for the Thailand samples were not available because only DNA samples and molecular results were collected. In Korea, hematological data from complete blood count tests were collected using the fluorescent flow cytometry method on the Sysmex XE‐2100 (Sysmex Corporation, Kobe, Japan) while maintaining anonymity. The study was conducted in accordance with the principles of the Declaration of Helsinki and was approved by the Institutional Review Boards of Jang‐won Medical Foundation (Seoul, Republic of Korea, IRB_2017010_MU01, IRB_2022044_MU01), with an exemption for written informed consent as the study used anonymized data.

### 2.2. DNA Extraction and Cloning

Total genomic DNA was extracted from the samples using the QIAamp DNA Blood Mini Kit (Qiagen, Valencia, CA, USA) according to the manufacturer’s instructions. Thereafter, the purity of the extracted DNA (OD 260/280 and OD 260/230) was determined using NanodropOne (Thermo Fisher Scientific, Madison, WI, USA).

Cloned PCR amplicons or synthesized genes for each mutation or wild‐type gene were used as positive controls, and the deletion region was confirmed based on data from the Globin Gene Server. The *HBAP2* gene was cloned into vector pMD20T, ‐‐^SEA^ mutation into pBT7‐C‐His, ‐‐^MED^ mutation into pBT7‐N‐GST, and all other types, including *HBA2* and *ABL1,* into pBHA. The nucleotide sequences were determined using Sanger sequencing and analyzed via gel electrophoresis. All cloned vectors harbored an ampicillin resistance gene. Subsequently, the plasmid DNA was transformed into NEB 5‐alpha Competent *Escherichia coli* (DH5α strain, NEB, Ipswich, MA, USA). After culturing the transformed *E. coli* on an LB‐ampicillin agar plate, the plasmid was extracted and used as the positive control.

### 2.3. Multiplex PCR and Gel Electrophoresis

**Figure 1:**
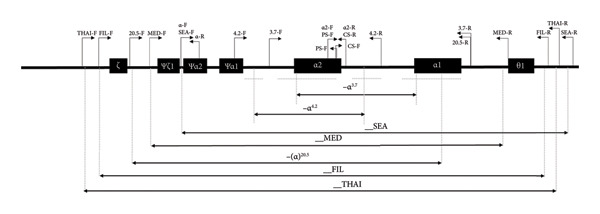
Schematic representation of the α‐globin gene cluster and primers used in the newly developed Kit. The filled boxes represent α‐globin genes with traditional gene annotation. The most common α‐thalassemia deletions are represented using black double‐headed arrows indicating the length of the deletion and annotation.

Multiplex PCR was performed using a final sample volume of 25 μL in 0.2‐mL thin‐walled tubes. The reaction mix contained 200 μM dNTPs, 1.25 U Hot Taq DNA polymerase, 20 mM Tris‐HCl, 100 mM KCl, 0.2 mg/mL BSA (bovine serum albumin), 4 mM MgCl_2_, 5% DMSO, 0.75 M betaine, 40 mM TMAC, 50–100 ng genomic DNA, and an optimized concentration of primers. The primers used are listed in Table 1; α/SEA‐F, α/PS‐F, and a‐CS‐R primer are written twice to clarify the target‐matched primer set. *ABL1* (NC_000009.12) was used as the internal control for Kit B. Further, the adjustment of relative concentrations was necessary for successful amplification using other Taq polymerases. The primers were either newly designed or were modified from previously reported primers. A schematic representation of the α‐globin gene cluster is shown in Figure 1 [4, 28]. Amplification for Kit A, B, and C was performed with an initial heat activation step of 15 min at 95°C, followed by 35 cycles of 95°C for 1 min, 65°C for 1 min, and 72°C for 2 min 30 s, with a final extension step at 72°C for 10 min, using the SimpliAmp Thermal Cycler (Life Technologies, Carlsbad, CA, USA).

**Table 1:** Information on the primer sequences used in this study.

KIT	Name	Sequence (5′ ⟶ 3′)	Target detection	Concentration (μM)	Amplicon size (bp)
A	α/SEA‐F	CTC​TGT​GTT​CTC​AGT​ATT​GGA​GGG​AAG​GAG	ψα2 gene	0.15	1010
	α‐R	TGA​AGA​GCC​TGC​AGG​ACC​AGG​TCA​GTG​ACC​G		0.15	
	α/SEA‐F	CTC​TGT​GTT​CTC​AGT​ATT​GGA​GGG​AAG​GAG	‐‐^SEA^deletion	0.15	704
	SEA‐R	ATA​TAT​GGG​TCT​GGA​AGT​GTA​TCC​CTC​CCA		0.15	
	FIL‐F	AAG​AGA​ATA​AAC​CAC​CCA​ATT​TTT​AAA​TGG​GCA	‐‐^FIL^deletion	0.30	550
	FIL‐R	GAG​ATA​ATA​ACC​TTT​ATC​TGC​CAC​ATG​TAG​CAA		0.30	
	THAI‐F	CAC​GAG​TAA​AAC​ATC​AAG​TAC​ACT​CCA​GCC	‐‐^THAI^deletion	0.15	411
	THAI‐R	TGG​ATC​TGC​ACC​TCT​GGG​TAG​GTT​CTG​TAC​C		0.15	

B	3.7‐F	CCC​CTC​GCC​AAG​TCC​ACC​C	‐α^3.7^deletion	0.20	2024
	3.7‐R	TCA​AAG​CAC​TCT​AGG​GTC​CAG​CGT​T		0.20	
	4.2‐F	CCC​CTG​ACT​CTC​TCT​CCA​CA	‐α^4.2^deletion	0.60	1480
	4.2‐R	AGG​TGG​GTT​TAC​AGG​CAT​GG		0.30	
	20.5‐F	GCC​CAA​CAT​CCG​GAG​TAC​ATG	‐‐^20.5^deletion	0.20	1007
	20.5‐R	AAA​GCA​CTC​TAG​GGT​CCA​GCG		0.20	
	MED‐F	TAC​CCT​TTG​CAA​GCA​CAC​GTA​C	‐‐^MED^deletion	0.20	807
	MED‐R	TCA​ATC​TCC​GAC​AGC​TCC​GAC		0.20	
	ABL‐F	CGA​GTC​TGG​TTG​ATG​CTG​TGA	ABL gene	0.40	330
	ABL‐R	CTA​GAG​ATG​GCG​AGG​CAC​AG		0.40	

C	α/PS‐F	GGGTCGAGGGGCGAGATG	α2‐globin gene	0.20	404
	α/CS‐R	CCA​TTG​TTG​GCA​CAT​TCC​GG		1.00	
	α/PS‐F	GGGTCGAGGGGCGAGATG	HbPS	0.60	266
	PS‐R	ACG​GCT​ACC​GAG​GCT​CCA​GCA			
	CS‐F	GCT​GAC​CTC​CAA​ATA​CCG​TC	HbCS	1.30	180
	α/CS‐R	CCA​TTG​TTG​GCA​CAT​TCC​GG			

After amplification, 5 μL of the product was electrophoresed on 2% agarose gel, which was prepared using 2X Tris‐acetate‐EDTA gel and stained with ethidium bromide, at 200 V for 15 min. After electrophoresis, the gel was visualized using an ultraviolet transilluminator.

### 2.4. MLPA

The MLPA assay was performed concurrently using the SALSA MLPA kit P140‐C1 HBA (MRC‐Holland, Amsterdam, the Netherlands) according to the manufacturer’s instructions. Specifically, the kit is designed to detect large deletions and duplications in one or more *HBA* exons and contains 34 probes for different *HBA* mutations and 11 reference probes. The amplified products were analyzed using the ABI 3730XL Genetic Analyzer (Applied Biosystems, Foster City, CA, USA). Further, MLPA data were analyzed using GeneMarker software v.1.91 (SoftGenetics, State College, PA, USA).

### 2.5. Sequencing

The PCR products were sequenced to determine each deletion type using the ABI 3730XL DNA analyzer (Applied Biosystems). Thereafter, the sequences obtained were analyzed using Sequence Scanner Software v2.0 (Applied Biosystems). The target‐specific primer sets for each mutation type were used. Further, the sequencing chromatograms obtained were compared to that of the reference α‐globin gene cluster sequence (GenBank accession number AE006462.1).

### 2.6. Validation of the Newly Developed Method

Analytical sensitivity, specificity, and precision measurements were realized to assess the performance of the newly developed PCR‐based method. Analytical sensitivity was evaluated by determining the limit of detection (LoD) for the cloned plasmid DNA with each target mutation, while analytical specificity was determined for patient samples with hemoglobinopathy, whose symptoms could be confused with those of α‐thalassemia. The intraday (four runs per day) and interday precision (20 different days) at different concentrations of cloned plasmid DNA with target mutations were assessed. Further, the intra‐ or interperson, equipment, and lot precision were also assessed (two runs per day and on three different days for person and equipment, four runs per day, and five different days for lot precision).

To assess the clinical performance of the newly developed method, diagnostic sensitivity and specificity were evaluated. The results of multiplex PCR performed for samples obtained in Korea were compared with those for samples obtained from Thailand as well as with the results of Sanger sequencing. Cohen’s kappa agreement values with 95% confidence intervals were calculated to compare the detection rates of *HBA* deletion mutations between the multiplex PCR and other assays. Kappa values were interpreted as follows: 0–0.20, slight; 0.21–0.40, fair; 0.41–0.60, moderate; 0.61–0.80 substantial; and 0.81–1, nearly perfect agreement [30]. Statistical analyses were performed using SPSS software v20.0 (SPSS Inc., Chicago, IL, USA) and MedCalc software (MedCalc, Ostend, Belgium).

## 3. Results

### 3.1. Multiplex PCR and Gel Electrophoresis

**Figure 2:**
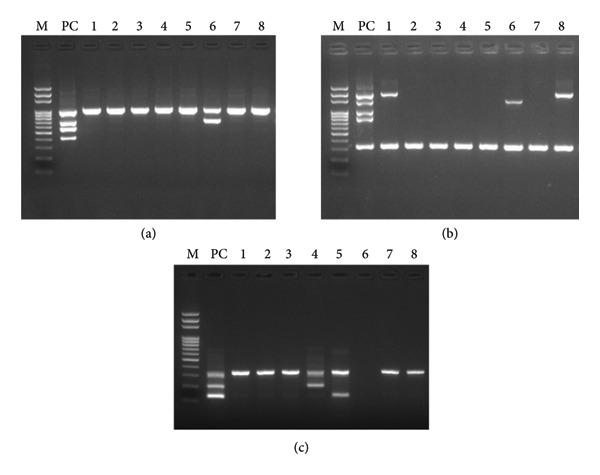
Gel images showing the results of multiplex PCR using the newly developed KIT. Representative results from the DNA samples of various α‐globin genotypes. M, 100 bp DNA ladder (Genomic Base); PC, positive control of each kit; 1–8, human samples. (a) KIT A (U2C001) shows one SEA‐type positive sample (Sample 6). (b) KIT B (U2C002) shows two samples positive for the 3.7 type (samples 1 and 8) and one positive for the 4.2 type (sample 6). The lowest amplification band (330 bp) corresponds to housekeeping gene (*ABL1*), used as an Internal control for this method. (c) KIT C (U2C003) shows samples positive for the PS type (sample 4) and the CS type (sample 5). Sample 6 is heterozygous for both the SEA and 4.2 types; therefore, no band is observed using KIT C due to the absence of *HBA2* genes. All positive types in this electrophoresis image are heterozygous for their respective type, and all possible types include homozygous and heterozygous as shown in the schematic figure (Supporting figure (available here)).

The number of alleles with α‐globin gene mutations in samples from Thailand was 851 (33.7%, 851/2522), and the proportions of −α^3.7^, ‐‐^SEA^, α^CS^α, −α^4.2^, α^PS^α, and ‐‐^THAI^ mutation types were 468 (18.6%), 199 (7.9%), 140 (5.6%), 25 (1.0%), 18 (0.7%), and 1 (0.04%), respectively. For the samples collected in Korea, multiplex PCR was performed using the newly developed kit (Figure 2), and the results obtained were compared with those based on Sanger sequencing. The results showed that the total number of alleles with α‐globin gene mutations was 852 (33.8%), while the number of alleles with −α^3.7^‐, ‐‐^SEA^‐, α^CS^α‐, α^PS^α‐, –^THAI^‐, and ‐‐^THAI^‐type mutations was 468 (18.6%), 199 (7.9%), 140 (5.6%), 26 (1.0%), 18 (0.7%), and 1 (0.04%), respectively. The agreement rate between the data corresponding to individuals in Thailand and that obtained in the present study was 99.92%. One case of mismatch was identified as a compound heterozygote (−α^3.7^/−α^4.2^). Further, only −α^3.7^ was detected in the samples from Thailand (Tables 2 and 3).

**Table 2:** Comparison of α‐thalassemia alleles among regions detected using two diagnostic kits.

Ethnic group	α‐Globin genotype	Alpha gene alleles			
		Newly developed KIT N (%)		In‐house KIT in Thailand N (%)	
Thailand	Negative	1670	(66.3%)	1671	(66.3%)
	SEA type	199	(07.9%)	199	(07.9%)
	THAI type	1	(00.0%)	1	(00.0%)
	FIL type	0	(00.0%)	0	(00.0%)
	20.5 type	0	(00.0%)	0	(00.0%)
	MED type	0	(00.0%)	0	(00.0%)
	3.7 type	468	(18.6%)	468	(18.6%)
	4.2 type	26^a^	(01.0%)	25	(01.0%)
	CS	140	(05.5%)	140	(05.5%)
	PS	18	(00.7%)	18	(00.7%)
Total		**2522**	(100.0%)	**2522**	(100.0%)

*Note:* The table shows the alpha‐thalassemia alleles of the samples that were tested using the newly developed Kit. It also shows data for Thailand. Bold values represent the overall total.

^a^Mismatch case observed in Thailand but not in Korea.

**Table 3:** Genotype frequencies of the alpha‐globin gene cluster between two diagnostic kits.

Mutation type	α‐Globin genotype	Newly developed KIT *N* (%)		In‐house KIT in Thailand *N* (%)	
Normal	αα/αα	560	(44.4%)	560	(44.4%)

Deletional	−α/αα	278	(22.0%)	279^a^	(22.1%)
	−α/−α	79	(6.3%)	78	(06.2%)
	−/αα	160	(12.7%)	160	(12.7%)
	−/−α	27	(2.1%)	27	(02.1%)
	−/−	0	(0.0%)	0	(00.0%)

Nondeletional	α^ND∗^α/αα	112	(08.9%)	112	(08.9%)
	α^ND^α/α^ND^α	1	(00.1%)	1	(00.1%)

Complex	−α/α^ND^α	31	(02.5%)	31	(02.5%)
	−/α^ND^α	13	(01.0%)	13	(01.0%)

Total		**1261**	(100.0%)	**1261**	(100.0%)

*Note:* The table shows the genotype frequencies of the samples that were tested using the newly developed Kit, as well as data from Thailand. There was one mismatch case each in the 4.2 type (Thailand) and 3.7 and 4.2 types (Korea). Bold values represent the overall total.

^a^ND; nondeletional.

Samples from 560 non‐Korean individuals living in Korea were collected for this study. Among them, 33 (5.89%) were identified as healthy carriers of α‐thalassemia given that their blood count results as well as their hemoglobin levels showed no significant variations. Among the healthy carriers, 22 (3.93%), 8 (1.43%), 2 (0.36%), and 1 (0.18%) were identified as harboring −α^3.7^‐, ‐‐^SEA^‐, α^CS^α‐, and −α^4.2^‐type mutations, respectively. To determine the proportion of α‐thalassemia, regardless of whether anemia symptoms were present or not given that α‐thalassemia is listed as a rare disease in Korea, 300 samples corresponding to native Koreans were examined. Thus, we observed negative results for all the samples (Table 4).

**Table 4:** Genotype frequencies of the α‐globin gene cluster in Koreans and foreign residents in Korea.

Ethnic group	α‐Globin genotype	Newly developed KIT
South Korea^a^	Negative	300
	SEA type	0
	THAI type	0
	FIL type	0
	20.5 type	0
	MED type	0
	3.7 type	0
	4.2 type	0
	CS	0
	PS	0
	Total	**300**

Foreign residents in Korea^b^	Negative	527
	SEA type	8
	THAI type	0
	FIL type	0
	20.5 type	0
	MED type	0
	3.7 type	22
	4.2 type	1
	CS	2
	PS	0
	Total	**560**

Total		**860**

Bold values represent the overall total.

^a^No compound‐heterozygotes were detected.

^b^Foreigners residing in Korea. Given that the samples were anonymized, it was not possible to identify the ethnic group; however, the group excludes Koreans.

Few hematologic features were noted in the 33 healthy carriers among non‐Korean individuals living in Korea. Further, among the nine samples that showed low Hb levels, four (4/22, 18.1%) were identified as harboring −α^3.7^‐type mutations and five (5/8, 62.5%) as harboring the ‐‐^SEA^ type. Therefore, the ‐‐^SEA^ type may be more severe than the 3.7 type. A similar phenomenon was observed with respect to the mean corpuscular volume (MCV) and mean corpuscular hemoglobin (MCH). Only 9.09% (2/22) of −α^3.7^‐type positive individuals showed low MCV and MCH levels, while 100% (8/8) of ‐‐^SEA^–type positive individuals showed low MCV and MCH levels (Table 5).

**Table 5:** Hematologic features of 33 samples with deletion mutations in foreign residents living in Korea.

Sample ID	Type of mutation	WBC	RBC	Hb	MCV	MCH	MCHC
JD_005	3.7 type	12.6 H	4.8	13.6	N/A	N/A	N/A
JD_037	3.7 type	N/A	N/A	14.0	N/A	N/A	N/A
JD_062	3.7 type	7.0	5.2	13.2	78.4 L	25.7 L	32.7
JD_067	3.7 type	4.8	4.3	12.7	90.4	29.6	32.7
JD_072	3.7 type	6.0	5.3	14.4	N/A	N/A	N/A
JD_088	3.7 type	4.9	4.1 L	13.3	99.8	32.7	32.8
JD_093	3.7 type	8.6	5.4	15.0	86.0	27.6	32.0
JD_163	3.7 type	7.6	4.9	13.8	N/A	N/A	N/A
JD_212	3.7 type	5.1	4.8	13.5	87.6	28.2	32.2
JD_232	3.7 type	8.3	6.0 H	12.9	N/A	N/A	N/A
JD_240	3.7 type	5.6	4.5	12.6	87.0	28.2	32.4
JD_278	3.7 type	7.8	5.1	14.3	N/A	N/A	N/A
JD_358	3.7 type	15.9 H	4.3	10.4 L	N/A	N/A	N/A
JD_359	3.7 type	6.1	4.5	12.5	85.6	27.9	32.6
JD_363	3.7 type	N/A	N/A	12.2	N/A	N/A	N/A
JD_379	3.7 type	N/A	N/A	11.9 L	N/A	N/A	N/A
JD_382	3.7 type	N/A	N/A	15.5	N/A	N/A	N/A
JD_415	3.7 type	6.7	5.5	15.4	87.5	28.2	32.2
JD_443	3.7 type	6.2	4.5	11.1 L	78.4 L	24.7 L	31.5
JD_461	3.7 type	4.5	5.2	14.6	86.4	28.2	32.6
JD_527	3.7 type	6.8	4.2	13.1	93.4	31.0	33.2
JD_549	3.7 type	6.9	4.2	12.8 L	89.4	30.4	34.0
JD_076	4.2 type	3.9 L	4.8	13.2	84.2	27.4	32.5
JD_173	CS type	5.8	4.7	14.4	92.0	30.4	33.1
JD_395	CS type	8.5	5.6	14.5	81.6	26.0	31.9
JD_043	SEA type	N/A	N/A	12.3	N/A	N/A	N/A
JD_220	SEA type	7.4	5.5	11.8 L	69.4 L	21.3 L	30.7 L
JD_230	SEA type	5.8	6.8 H	14.1	67.2 L	20.8 L	31.0
JD_270	SEA type	11.2 H	5.2	11.0 L	68.3 L	21.0 L	30.7 L
JD_412	SEA type	9.4	5.4 H	11.2 L	67.6 L	20.6 L	30.4 L
JD_430	SEA type	8.9	6.0	14.1	75.9 L	23.7 L	31.2
JD_467	SEA type	8.9	5.5 H	11.8 L	72.6 L	21.7 L	29.9 L
JD_554	SEA type	4.4	4.8	10.7 L	69.7 L	22.3 L	32.0

*Note:* Reference range of each index: WBC (4.0∼10.0 × 10^3^/μL), RBC (Male‐4.2∼6.2 × 10^6^/μL, Female‐3.8∼5.4 × 10^6^/μL), Hb (Male‐13.0∼17.0 g/dL, Female‐12.0∼16.0 g/dL), MCV (80.0∼100.0 fl), MCH (26.0∼34.0 pg), MCHC (31.0∼36.0%). H, high; L, low determined based on the normal value of each hematological parameter.

Abbreviation: N/A, not applicable.

### 3.2. MLPA

One case was positive for −α^3.7^‐ and −α^4.2^‐type mutations; however, the collected data showed that this case was only positive for the −α^3.7^‐type mutation. Thus, to identify whether a mismatch occurred, we performed MLPA using 10 cases of each mutation type. The MLPA results showed a 100% match rate with the newly developed kit.

We also observed that −α^3.7^‐ and −α^4.2^‐type mutations had various deletion points and could be distinguished from other deletion types based on the length of the deletion sequences. Thus, MLPA could be used to distinguish these various mutation types using the A–F and A–C types for −α^3.7^ and −α^4.2^, respectively (Product Description version C1‐06, Issued 05 April 2023). Further, in this study, the D type was dominant in the −α^3.7^ type, while the F and A types were rare. For the −α^4.2^‐type mutation, the C type was dominant, while the D type was rarely detected (data not shown).

### 3.3. Sequencing

**Figure 3:**
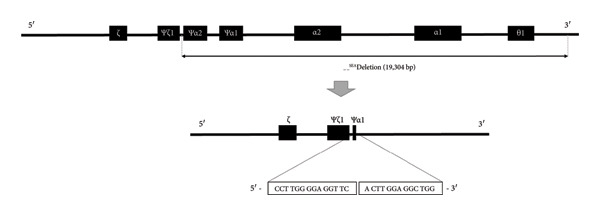
DNA sequence of SEA‐type deletion in a Thai sample. The location of the break in the chromosome of the Thai sample was NG_000006.1:g.26260_45563del19304, which has not been uploaded into hemoglobin databases, and has only been reported in one study conducted in Mexico in 2006. The filled boxes represent α‐globin genes with traditional gene annotation, and the ‐‐^SEA^ deletion type is indicated using black double‐headed arrows, which are indicative of the length of the deletion and annotation. The image shows an enlarged breakpoint, and the sequence enclosed in a rectangle was obtained from the electropherogram.

Sanger sequencing was performed using 701 positive cases, and a match rate of 99.86% (700/701) was obtained. The deletion points were exactly matched for most types; however, for the ‐‐^SEA^ type, the point was different for the HbVar or The National Center for Biotechnology Information (NCBI) databases. The breakpoint of the ‐‐^SEA^ type, which was previously characterized, ranges from c.165402 to c.184701 on chromosome 16 [31–35]. In this study, the deletion start and end points were c.165397 and c.184700 on chromosome 16, respectively (Figure 3), consistent with the results of a previous study [5]. Based on this previous report, the mismatch case could be considered as a ‐‐^SEA^‐type mutation and the agreement rate was 100%. For a more precise diagnosis and advanced research, these mutations need to be added to databases.

### 3.4. Validation of the Newly Developed Method

We evaluated the analytical performance of the newly developed method, including analytical sensitivity, specificity, and precision (Table 6). The analytical sensitivity was 95%–100% (19/20 or 20/20 each) at each LoD concentration. The analytical specificity was 100%, as no detections occurred in any of the 15 hemoglobinopathy samples. Finally, analytical precision was confirmed in two aspects: intra‐ and interday repeatability and intra‐ or interperson, equipment, and lot‐to‐lot reproducibility. The intra‐and interday repeatability was 96.3%–100% (77/80–80/80), while reproducibility across individuals, equipment, and lots was 100%.

**Table 6:** Validation results for the newly developed diagnostic method.

Validation set	SEA	FIL	THAI	3.7	4.2	20.5	MED	PS	CS
Analytical performance									
Sensitivity^a^	95% (19/20)	95% (19/20)	100% (20/20)	100% (20/20)	95% (19/20)	100% (20/20)	95% (19/20)	95% (19/20)	100% (20/20)
Specificity^b^	100% (15/15)	100% (15/15)	100% (15/15)	100% (15/15)	100% (15/15)	100% (15/15)	100% (15/15)	100% (15/15)	100% (15/15)
Precision									
Repeatability^c^	98.8% (79/80)	98.8% (79/80)	96.3% (77/80)	98.8% (79/80)	100% (80/80)	98.8% (79/80)	97.5% (78/80)	97.5% (78/80)	98.8% (79/80)
Reproducibility									
Person	100% (12/12)	100% (12/12)	100% (12/12)	100% (12/12)	100% (12/12)	100% (12/12)	100% (12/12)	100% (12/12)	100% (12/12)
Equipment	100% (12/12)	100% (12/12)	100% (12/12)	100% (12/12)	100% (12/12)	100% (12/12)	100% (12/12)	100% (12/12)	100% (12/12)
Lot	100% (60/60)	100% (60/60)	100% (60/60)	100% (60/60)	100% (60/60)	100% (60/60)	100% (60/60)	100% (60/60)	100% (60/60)
Clinical performance									
Sensitivity	100% (90/90)	100% (41/41)	100% (41/41)	98.9% (89/90)	100% (43/43)	100% (40/40)	100% (40/40)	100% (43/43)	100% (90/90)
Specificity	100% (334/334)	100% (383/383)	100% (384/384)	100% (375/375)	100% (422/422)	100% (425/425)	100% (425/425)	100% (343/343)	100% (296/296)

^a^Analytical sensitivity is evaluated at each limit of detection (LoD) concentration.

^b^Analytical specificity is evaluated using 15 hemoglobinopathy‐positive samples other than alpha‐thalassemia. The results 15/15 mean no positive bands for all 15 samples.

^c^Repeatability is evaluated in 50x, 25x, 10x, 3x, 1x LoD, and results for all concentrations except 1x LoD were 100%.

Clinical sensitivity and specificity were confirmed by comparing the results obtained in this study and MLPA data. Since the Hb PS‐type mutation was not included in the MLPA P140‐C1 panel, Sanger sequencing was performed. The sensitivity and specificity for ‐‐^SEA^, ‐‐^THAI^, −α^4.2^, α^CS^α, and α^PS^α types were all 100%, and the Cohen’s kappa value was 1.0. For the −α^3.7^‐type mutation, the sensitivity and specificity were 98.9% and 100%, respectively, and the Cohen’s kappa value was 0.996.

## 4. Discussion

In this study, we developed a conventional PCR method for the diagnosis of α‐thalassemia that is fast, less labor‐intensive, and cost‐effective. The previously developed diagnostic kit for α‐thalassemia uses a PCR‐based hybridization method, which is more labor‐intensive and time‐consuming, with sensitivity and specificity at 96.67% and 92.37%, respectively [21, 28, 29, 36–38]. The newly developed method had a sensitivity and specificity of 99.53% and 100%, respectively, indicating excellent analytical performance. Further, we analyzed the analytical performance of our method using all mutation types, and discrepancies were observed only for one mutation type, particularly, the sample with both −α^3.7^‐ and −α^4.2^‐type mutations, which showed similar band sizes. The commercial kits using the PCR‐based hybridization method is not allowed for in vitro diagnostic purpose in Korea. Additionally, these kits require an additional device for the hybridization step; therefore, we were unable to compare the test results. Instead, we tested approximately 700 samples to compare our method with Sanger sequencing and MLPA, which are currently used for α‐thalassemia diagnosis in Korea. Confirmatory tests using MLPA revealed that detection using our kit was accurate. Cross‐reactivity tests on samples of other blood diseases that showed similar symptoms and required differential diagnoses showed no cross‐reaction; only the target mutation was detected with high accuracy.

Sanger sequencing was performed using the positive cases, and a different breakpoint for the ‐‐^SEA^ mutation was detected. This deletion point was identified as c.165397–c.184700, consistent with previous reports, but differed from those proposed in the HbVAR or NCBI databases [35]. As most of the positive samples in this study were geographically confined to Thailand, determining which one was correct was challenging. The ‐‐^SEA^‐type break point described in this study is rarely reported globally. This is the first study to identify this mutation type in Korea. Therefore, it is necessary to record both deletion points such as for −α^3.7^‐ and −α^4.2^‐type mutations in databases.

Numerous studies on the prevalence of α‐thalassemia have been published. In this study, we performed a comparative analysis of 15 studies to confirm whether the results obtained for the Thailand samples were similar to those of our meta‐study (Table S1). Most of the collected samples were from Thailand; therefore, the prevalence of α‐thalassemia in this region was the focus of this study. Further, given that the distribution of α‐thalassemia varies by region; seven reports from other countries were included. All the mutations were classified under nine types, and the most prevalent types were analyzed. In Thailand studies (Group 1), ‐‐^SEA^‐type mutations were the most dominant (16.6%) followed by −α^3.7^‐ and α^CS^α‐type mutations (12.5% and 7.2%, respectively) [12, 22, 39–44]. Five studies from Southeast and East Asia showed similar results: the predominance of ‐‐^SEA^‐, −α^3.7^‐, and α^CS^α‐type mutations were 10.1%, 9.1%, and 1.7%, respectively (Group 2) [21, 23, 45, 46]. However, two cases in Italy showed very different orders of dominant types (Group 3), with −α^3.7^ mutations being the most dominant type (25.2%) followed by others (8.0%) and ‐‐^MED^ (5.4%) mutation types [19, 20]. Further ‐‐^THAI^ and ‐‐^FIL^ mutation types were rarely observed in Groups 1 and 2; they were mostly observed in a study in Malaysia. The −(α)^20.5^‐type mutations were also rarely observed in Group 3. Similar to a previous report, ‐‐^SEA^‐type mutations are common in Southeast Asia, while ‐‐^MED^ types are common in Europe, and the −α^3.7^ and −α^4.2^ types are common in both regions [3]. In this study, ‐‐^SEA^, −α^3.7^, and −α^4.2^ mutation types accounted for 49.3% of the samples from Thailand, similar to the observation made for Group 1 [3].

Samples from Thailand, Korea, and foreign residents in Korea were tested for α‐thalassemia, and the prevalence of the condition within each group was examined. Samples from Korea and foreign residents in Korea were examined to identify the proportion of mutation carriers in a population without any symptoms that may be overlooked due to the low prevalence of this disease. As expected, samples from Thailand showed the highest proportion (55.6%) of carriers, with ‐‐^SEA^, −α^3.7^, and α^CS^α being the most prevalent mutation types. Foreign residents in Korea constitute 5.8% of the population in Korea, and most of them harbored the −α^3.7^ mutation type. Meanwhile, all Korean samples showed negative results. However, there were differences in the sample population given that samples were collected from suspected cases in Thailand, while random sampling was performed for asymptomatic Koreans and other foreigners residing in Korea. Thus, it is almost impossible to detect α‐thalassemia in Koreans. However, with global population shifts, α‐thalassemia is no longer confined to certain regions [24, 25]. Due to the increase in the number of immigrants, the population of foreign residents living in Korea and that of second‐generation immigrants are increasing. This shift indicates that α‐thalassemia can occur not only in Korea, but in any region worldwide [26, 27, 47, 48]. Therefore, the diagnosis of α‐thalassemia is necessary in Korea, as the prevalence may not be as low as expected. Additionally, guidelines for blood transfusion are also needed, and a new carrier detection test for α‐thalassemia should be established [49, 50]. It is also worth noting that this is the first report on the prevalence of α‐thalassemia in Korea that confirms the associated mutation types in detail.

Given that we randomly collected residual samples, there were some limitations to interpreting the correlation between hematologic features and molecular test results. Our results showed that more severe mutations are characterized by more serious pathological features, although patients do not complain of symptoms. Research is limited to northern Thailand, and clinical data are insufficient owing to the anonymization of all samples in this study. However, it is noteworthy that the prevalence data were obtained by examining domestic residents in South Korea. Further, α‐thalassemia is a serious and life‐threatening disease, depending on its severity, and it may go undetected during the life of a patient. However, its diagnosis is costly and inaccessible in areas with a high prevalence rate. This should not be the case. Therefore, the rapid, easy‐to‐analyze, and inexpensive diagnostic method developed in this study can be a useful tool for the diagnosis of α‐thalassemia.

Large deletion mutations such as ‐‐^KOL^, ‐‐^BRIT^, ‐‐^CAL^, and ‐‐^DutchI^ have lost gene clusters comparable in size to those of ‐‐^THAI^, ‐‐^FIL^, and ‐‐^SEA^ types. Although these mutations have similar deletion sizes or ranges of deleted gene clusters, they could not be detected using our method or exhibited different amplicon sizes than our target in the case of ‐‐^KOL^. In most cases, there is a possibility that one of the primers for ‐‐^SEA^, ‐‐^FIL^, ‐‐^THAI^, ‐‐^MED^, and −(α)^20.5^ can bind to ‐‐^BRIT^, ‐‐^CAL^, and ‐‐^DutchI^ positive samples, but not in both forward and reverse direction. Furthermore, the expected PCR product size is too large to be amplified, preventing detection or differentiation between these mutations. For detectable mutation other than our target, the amplicon size will vary due to different breakpoint positions and deletion sizes. For instance, the mutation ‐‐^KOL^ can be detected with the FIL‐forward and SEA‐reverse primer, yielding a 500‐bp amplicon. If this new method is applied in areas with high prevalence of ‐‐^KOL^, further differential diagnosis will be required when an amplicon in the 500–550‐bp range is detected in KIT A.

In conclusion, we successfully developed a rapid, easy‐to‐use, and cost‐effective diagnostic method for α‐thalassemia diagnosis. The novel kit showed performance comparable to those of other detection kits. Thus, its application can considerably facilitate the detection of α‐thalassemia. Therefore, the findings of this study can be applied in the development of diagnostic tools for other hereditary diseases.

**Nomenclature**
Hb: Hemoglobin
Hb CS: Hb Constant Spring
Hb PS: Hb Paksé
MCH: Mean corpuscular hemoglobin
MCV: Mean corpuscular volume
MLPA: Multiplex ligation‐dependent probe amplification

## Consent

The informed consent has been waivered by the Institutional Review Boards as the study used anonymized data.

## Disclosure

All the authors have read and approved the final manuscript. The company had no role in the study design, data collection and analysis, decision to publish, or manuscript preparation.

## Conflicts of Interest

The authors declare no conflicts of interest.

Some of the authors are employees of U2Bio Co. Ltd., which provided funding and laboratory resources for this research.

## Author Contributions

Si Nae Park​ performed the experiments and contributed to the writing of the manuscript. Jin Roh and Jin‐Tae Kim contributed to the collection of samples and corresponding data. Si Nae Park and Min‐Jung Song confirm the authenticity of all the raw data.

## Funding

The study was supported by U2Bio Co. Ltd., Seoul, Korea.

## Supporting Information

Additional supporting information can be found online in the Supporting Information section.

## Supporting information


**Supporting Information 1** Supporting Figure. The schematic figure of electrophoresis of alpha‐thalassemia positive‐type detectable using this method.

**Supporting Information 2** Supporting Table. Alpha‐thalassemia alleles reported in other studies.

**Supporting Information 3** The present study was approved by the Institutional Review Boards of Jang‐won Medical Foundation (Seoul, Republic of Korea) (IRB_2017010_MU01, IRB_2022044_MU01), and the requirement for written informed consent was waived as the study used anonymized data.

## Data Availability

The datasets generated and/or analyzed during the current study are available from the corresponding author upon reasonable request.
