# Supplementary material for: Diagnostic Utility of a Multiplex PCR Assay in Detecting Common Mutations of the α‐Globin Gene in α‐Thalassemia
Source: Anemia. 2025 Oct 14;2025:9991675. doi: 10.1155/anem/9991675 (PMC12539667; doi:10.1155/anem/9991675)

Supplemental Figure

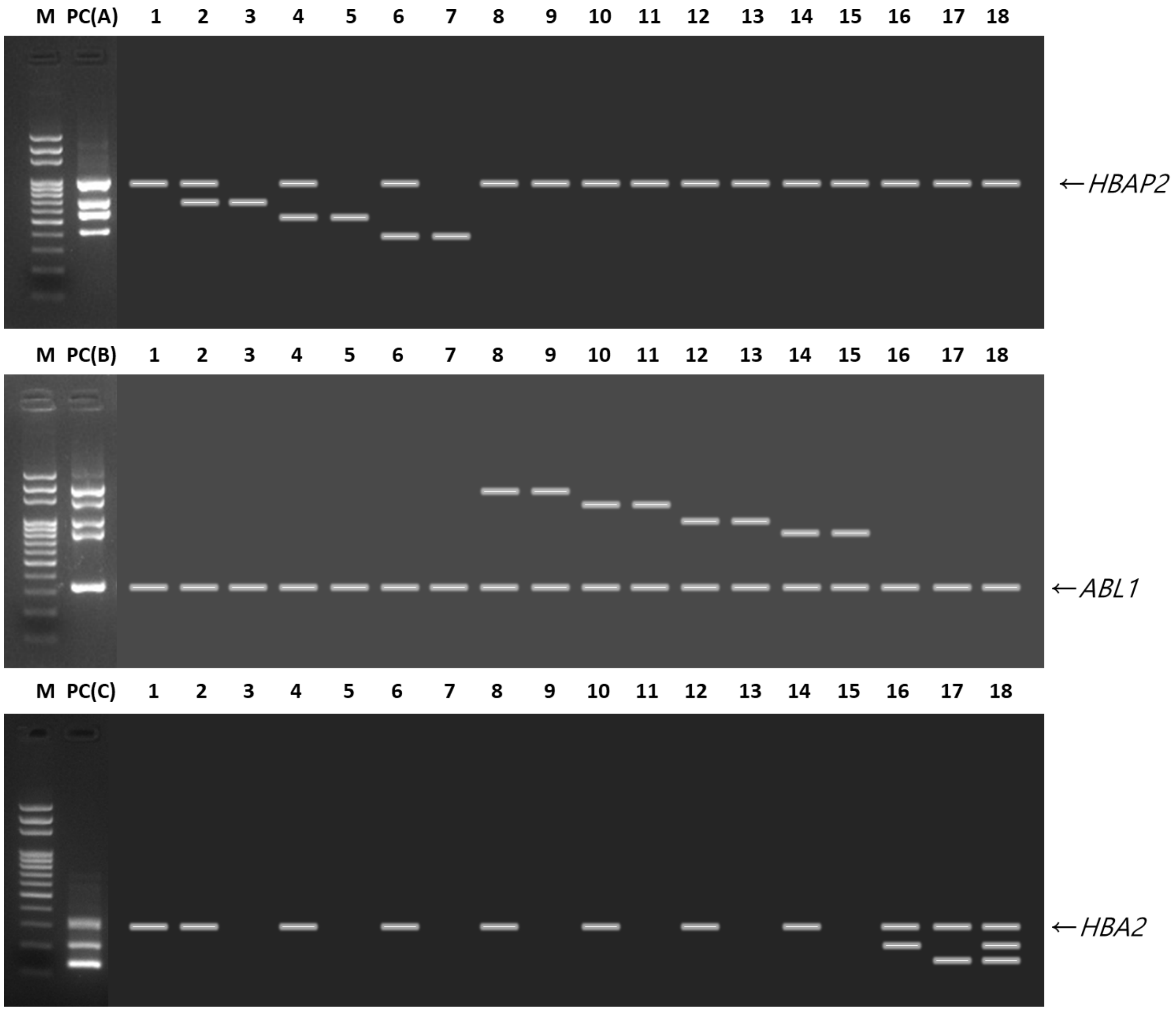

Supplemental Figure. The schematic figure of electrophoresis of alpha-thalassemia positive type detectable using this method.

The presence or absence of HBA2 amplification bands determined between heterozygous and homozygous for each mutation type. PS type and CS type cannot be distinguished whether heterozygous or homozygous unless they have compound heterozygous with other types of mutation. Other 7 positive types could be distinguished whether heterozygous or homozygous. M, 100 bp DNA ladder (Genomic Base); PC(A), positive control of Kit A; PC(B), positive control of Kit B; PC(C), positive control of Kit C. 1, Negative for all type of alpha-thalassemia; 2, SEA heterozygous type; 3, SEA homozygous type; 4, FIL heterozygous type; 5, FIL homozygous type; 6, THAI heterozygous type; 7, THAI homozygous type; 8, 3.7 heterozygous type; 9, 3.7 homozygous type; 10, 4.2 heterozygous type; 11, 4.2 homozygous type; 12, 20.5 heterozygous type; 13, 20.5 homozygous type; 14, MED heterozygous type; 15, MED homozygous type; 16, PS-positive type; 17, CS-positive type; 18, PS/CS compound heterozygous type.

Figure 3 – editable raw image

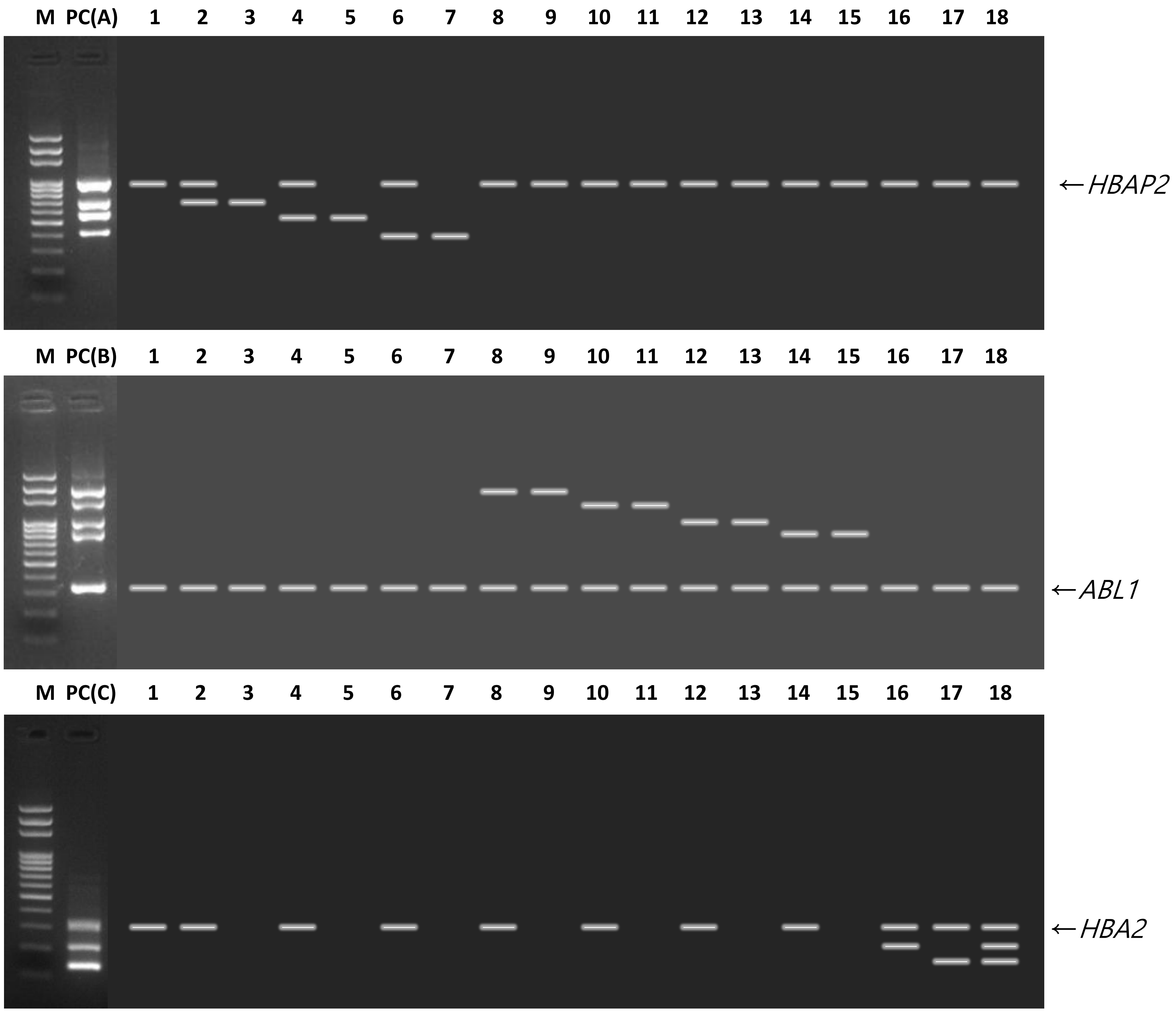

Supplement: Supplementary file 1 — Supporting Information 1 Supporting Figure. The schematic figure of electrophoresis of alpha‐thalassemia positive‐type detectable using this method. [file ANEM-2025-9991675-s003.pdf]
