# Supplementary material for: Diagnostic Utility of a Multiplex PCR Assay in Detecting Common Mutations of the α‐Globin Gene in α‐Thalassemia
Source: Anemia. 2025 Oct 14;2025:9991675. doi: 10.1155/anem/9991675 (PMC12539667; doi:10.1155/anem/9991675)
Supplement: Supplementary file 2 — Supporting Information 2 Supporting Table. Alpha‐thalassemia alleles reported in other studies. [file ANEM-2025-9991675-s002.docx]

**Table S1. Alpha-thalassemia alleles reported in other studies.**

| **Regional Group** | **Author** | **Ref** | **Year** | **Ethnic group** | **Total** | **Neg.** | **SEA** | **THAI** | **FIL** | **20.5** | **MED** | **3.7** | **4.2** | **CS** | **PS** | **Other** | **N/A** |
| --- | --- | --- | --- | --- | --- | --- | --- | --- | --- | --- | --- | --- | --- | --- | --- | --- | --- |
| **Thailand -Group1** | G. Fucharoen | 37 | 2004 | Thailand | 602 | 441 | 10 | 0 | 0 | 0 | 0 | 123 | 6 | 22 | 0 | 0 | 0 |
|  | K. Sanchaisuriya | 38 | 2003 | Thailand | 404 | 251 | 39 | 0 | 0 | 0 | 0 | 87 | 4 | 21 | 2 | 0 | 0 |
|  | G. Fucharoen | 39 | 2006 | Thailand | 262 | 210 | 6 | 0 | 0 | 0 | 0 | 33 | 4 | 8 | 1 | 0 | 0 |
|  | N. Sae-ung | 40 | 2007 | Thailand | 486 | 178 | 242 | 1 | 0 | 0 | 0 | 7 | 0 | 42 | 5 | 11 | 0 |
|  | V. Viprakasit | 41 | 2009 | Thailand | 710 | 0 | 353 | 2 | 0 | 0 | 0 | 136 | 6 | 181 | 23 | 9 | 0 |
|  | V. Viprakasit | 42 | 2014 | Thailand | 700 | 652 | 21 | 0 | 0 | 0 | 0 | 18 | 6 | 3 | 0 | 0 | 0 |
|  | P. Lithanatudom | 12 | 2016 | Thailand | 282 | 248 | 5 | 0 | 0 | 0 | 0 | 26 | 0 | 3 | 0 | 0 | 0 |
|  | A. Chaibunruang | 20 | 2018 | Thailand | 700 | 574 | 11 | 0 | 0 | 0 | 0 | 88 | 3 | 19 | 5 | 0 | 0 |
| **Other country of Asia -Group2** | Ko TM | 43 | 1991 | Taiwan | 174 | 0 | 168 | 5 | 0 | 0 | 0 | 0 | 0 | 0 | 0 | 1 | 0 |
|  | W. Zhou | 44 | 2013 | China  (sample bank) | 1728 | 916 | 425 | 5 | 0 | 0 | 0 | 215 | 147 | 0 | 0 | 20 | 0 |
|  | W. Zhou | 44 | 2013 | China  (mass screen) | 6000 | 5704 | 140 | 3 | 0 | 0 | 0 | 110 | 35 | 0 | 0 | 0 | 8 |
|  | B. Rosnah | 19 | 2012 | Malaysia | 800 | 763 | 2 | 0 | 0 | 0 | 0 | 34 | 1 | 0 | 0 | 0 | 0 |
|  | R. Ahmad | 21 | 2013 | Malaysia | 10032 | 6894 | 1165 | 26 | 27 | 0 | 0 | 1338 | 111 | 316 | 0 | 155 | 0 |
| **Europe -Group3** | R. Origa | 17 | 2007 | Italy | 502 | 0 | 0 | 0 | 0 | 2 | 248 | 207 | 5 | 0 | 0 | 40 | 0 |
|  | R. Origa | 18 | 2014 | Italy | 7188 | 2492 | 2 | 0 | 0 | 4 | 168 | 1730 | 27 | 1 | 0 | 578 | 2186 |
| **Total alleles of alpha-globin** | | | | | 30570 | 19323 | 2589 | 42 | 27 | 6 | 416 | 4152 | 355 | 616 | 36 | 814 | 2194 |

Neg, Negative
