# Supplementary material for: Diagnostic Utility of a Multiplex PCR Assay in Detecting Common Mutations of the α‐Globin Gene in α‐Thalassemia
Source: Anemia. 2025 Oct 14;2025:9991675. doi: 10.1155/anem/9991675 (PMC12539667; doi:10.1155/anem/9991675)
Supplement: Supplementary file 3 — Supporting Information 3 The present study was approved by the Institutional Review Boards of Jang‐won Medical Foundation (Seoul, Republic of Korea) (IRB_2017010_MU01, IRB_2022044_MU01), and the requirement for written informed consent was waived as the study used anonymized data. [file ANEM-2025-9991675-s001.zip › IRB_2017010_MU01_Ethical_Approval.pdf]

IRB Approval Letter

We hereby notify you that the trial study you applied for below, was reviewed on 'Jangwon Medical Foundation Institutional Review Board (IRB)' in the year 2018, and the decision is as follows.

**Recipient**	**Institution**	**Investigator**	**Title/Position**
	U2Bio Co., Ltd.	Si Nae Park	Researcher
**Study Title**	Genotype analysis of 7 types of alpha-thalassemia on patients and normal individuals by using multiplex polymerase chain reaction (PCR)		
**Study Number**	CR_2017010		
**Approval Number**	IRB_2017010_MU01		
**Study classification**	☐Confirmatory clinical performance study ☐Exploratory clinical performance study<br>■Human Derivatives study		
**Deliberation method**	☐Full board review ■Expedited review ☐Others		
**Committee classification**	■Institutional Review Board (IRB) ☐Clinical Review Board (CRB)		
**Type of Review**	☐Initial Review ☐Continuing Review ■Amendment Review<br>☐Post-amendment expedited review ☐Post-amendment Full Board review<br>☐Study closure ☐Final report ☐Serious adverse event Report (SAERs)<br>☐Protocol violation (non-compliance) / deviation<br>☐IRB Review Exemption ☐Appeal of IRB Decision ☐Others		
**Risk Level**	■Minimal risk ☐Low risk ☐Moderate risk ☐High risk		
**Consent Acquisition Method**	☐Written consent ■Exemption from written consent ☐Not applicable		
**Number of Center**	■Single-center trial<br>☐Multicenter trial: Total Number of Institutions ( )		
**Study period**	From the initial approval date by the Institutional Review Board (IRB), which is 24 months (2017.08.30~2019.08.29)		
**Review Date**	June 18, 2018	**Review Site**	Committee room
**Approval Date**	June 19, 2018	**IRB Approval Expiration Date**	June 18, 2019
**Result**	■Approval ☐Qualified permission ☐Post-amendment full board review<br>☐Post-amendment expedited review ☐IRB Review Exempted<br>☐Rejected ☐Suspension		
**Comment**	This study has been **approved** by the Institutional Review Board (IRB).<br>This study on human-derived material subjects was approved as the value of the test was recognized, there were no ethical problems and scientifically valid in the protocol.		

Jangwon Medical Foundation Institutional Review Board
